# Persistent Postauricular Swelling in a Pediatric Patient: Unraveling the Cat Scratch Disease

**DOI:** 10.7759/cureus.88662

**Published:** 2025-07-24

**Authors:** Oluwasegun A Shoewu, Charly Oscanoa, Auda H Baker, Iyad Baker

**Affiliations:** 1 Family Medicine, Hackensack Meridian Health Palisades Medical Center, North Bergen, USA; 2 Family Medicine, Hackensack Meridian School of Medicine, Hackensack, USA

**Keywords:** bartonella henselae, cat scratch, lymphadenitis, postauricular, swelling

## Abstract

The etiology of posterior auricular swelling can be infectious, congenital, oncologic, or autoimmune. The most common cause of swelling in this region is reactive lymphadenopathy. Among the infectious etiologies, *Bartonella henselae* is an often-overlooked cause in immunocompetent individuals due to its varied clinical features. This swelling may fail to resolve despite the use of standard antibiotics, thereby presenting a diagnostic challenge for clinicians. We present a healthy nine-year-old male with no significant medical history who presented with persistent, painful postauricular swelling for 12 days following treatment for streptococcal pharyngitis. Despite a full course of antibiotics, the swelling progressed to a fluctuant mass with overlying redness. Further history revealed recent kitten exposure and a healed scratch on the right forearm, which raised suspicion of cat scratch disease (CSD). Comprehensive physical examination showed a hypopigmented healed scar on the right distal forearm and a posterior auricular abscess, which was drained and cultured. The diagnosis was confirmed by elevated *Bartonella henselae* serology with an IgM titer of 1:80 (reference range <1:20) and an elevated IgG antibody titer of 1:512 (reference range <1:64). Azithromycin was initiated due to serologic confirmation and the presence of suppuration, leading to resolution within three weeks. This case conclusively highlights the importance of considering CSD in children with unilateral lymphadenitis unresponsive to standard antibiotics, emphasizing the value of detailed history and physical examination in the primary care setting.

## Introduction

Cat scratch disease (CSD) is a zoonotic disease caused by the indolent bacterium, *Bartonella henselae* [1]. It has a worldwide distribution, affecting the pediatric and adult populations, being more prevalent in the former [2]. It is transmitted through the scratch or bite from infected cats, kittens, or fleas that harbor the bacteria. Salivary contact of infected cats with open human wounds can transmit the disease. In humans, the bacteria affect endothelial cells, causing a proinflammatory response, resulting in a local infection that manifests as regional lymph node enlargement in immunocompetent individuals [2].

It is a common cause of regional lymphadenopathy in children and young adults, often associated with a history of cat exposure and an inoculation lesion [1,2]. While most cases follow a benign and self-limited course [3], approximately 10-15% of patients may develop suppurative lymphadenitis requiring medical intervention [2]. Commonly affected lymph nodes include the cervical, submandibular, axillary, and inguinal nodes [2,4].

Other presentations of CSD include low-grade fever, malaise, and headache. In rare cases, systemic involvement may occur, including hepatic or splenic abscesses, chorioretinitis, Parinaud oculoglandular syndrome, osteomyelitis, pneumonia, endocarditis, and encephalitis [1,5-7]. The non-specific nature of CSD can make diagnosis challenging, particularly when the initial therapy fails. In such cases, detailed exposure history and careful physical examination with targeted imaging and serologic testing are critical for timely diagnosis and management.

This case underscores the need to maintain a high index of suspicion for CSD in children with persistent or unusual lymphadenopathy unresponsive to routine therapy.

## Case presentation

A previously healthy nine-year-old boy with no chronic illnesses presented with a 12-day history of progressive, painful swelling behind his right ear. The patient first presented with this swelling along with low-grade fever (maximum 100.5°F) and sore throat to an outside hospital. He had no allergies, surgical history, recent travel, sick contacts, or relevant family history. He was evaluated without detailed environmental risk factors, diagnosed with Group A *Streptococcus* pharyngitis, and prescribed a 10-day course of oral amoxicillin-clavulanate. The fever resolved within three days, but the postauricular swelling persisted with progressive enlargement.

On day 12, the swelling became more prominent, erythematous, and fluctuant, prompting emergency department evaluation. The patient did not have any sore throat at this time. Further history revealed that the patient was consistently exposed to a kitten for months at home.

On physical examination, the patient appeared well and afebrile. A crusted 2-3 mm papule was noted on the right temporal scalp. A hypopigmented linear scar on the distal right forearm was identified as a healed scratch from a kitten. There was a 2.5 cm fluctuant, tender, erythematous swelling on the right postauricular region. Other findings included sub-centimeter, shotty, mildly tender cervical and submandibular nodes. No pharyngeal erythema or exudate, ear discharge, or signs of mastoiditis were present. Otoscopic and cranial nerve examinations were normal.

**Figure 1:**
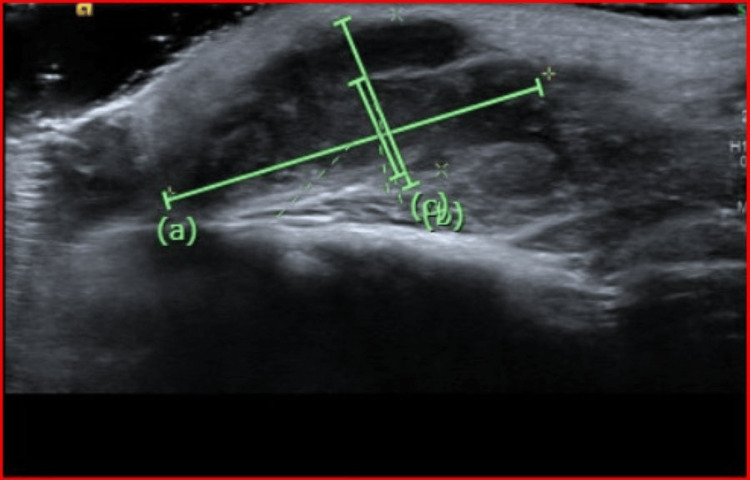
Ultrasound of the right posterior auricular region. Ultrasound of the right posterior auricular region showing abscess with measured dimensions of (a) 2.97 cm, (b) 1.36 cm, and (c) 0.78 cm within the right retroauricular lymphadenitis with extension into the adjacent musculature.

**Figure 2:**
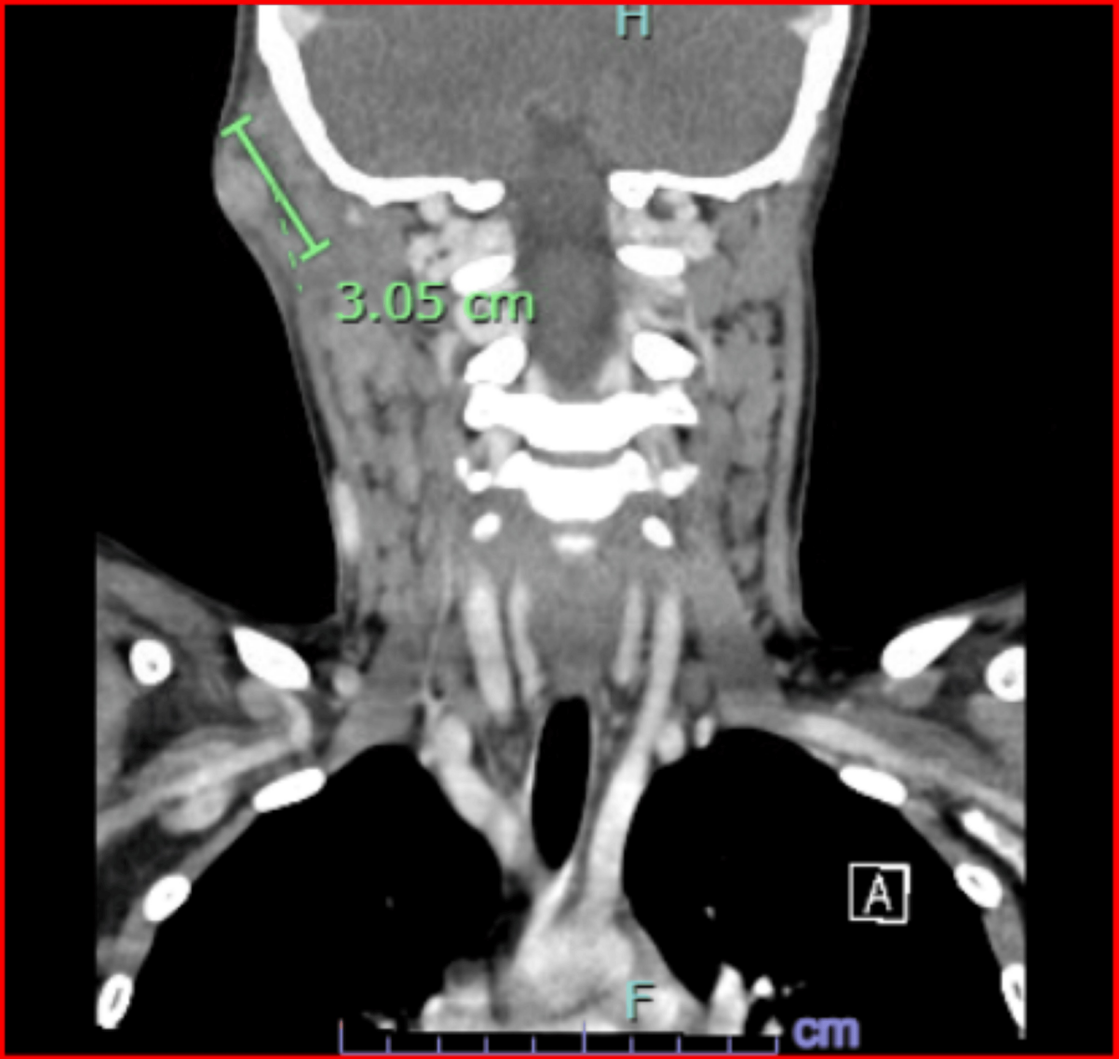
CT of the soft tissue neck with contrast. CT shows a mixed-attenuation soft tissue abnormality in the right posterior auricular area of the upper neck adjacent to the posterior margin of the mastoid bone that indicates suppurative or abscessed lymph node within the central low-density component measuring 3.05 cm in diameter. The mastoid air cells and middle ear cavities are clear. Additional enlarged lymph nodes are identified in the sub-adjacent right posterior triangle of the suprahyoid neck and mild lymphadenopathy in the left posterior triangle of the suprahyoid neck (not highlighted in the image).

Laboratory evaluation showed a normal complete blood count and basic metabolic panel. C-reactive protein (CRP) was within normal limits at 0.2 mg/dL (reference range <0.5 mg/dL). Blood cultures were negative. The QuantiFERON test returned negative. Ultrasound of the right posterior auricular region showed abscess formation within the right retroauricular lymphadenitis and extending into the adjacent muscle (Figure 1). Contrast-enhanced CT of the neck showed a mixed-attenuation soft tissue mass in the right postauricular region adjacent to the mastoid bone (Figure 2), consistent with a suppurative lymph node with intramuscular extension. There was no bony involvement or mastoiditis.

Due to clinical worsening despite routine antibiotics, the differential was expanded to include atypical bacteria (such as *B. henselae*, atypical mycobacteria), viral etiologies, deep neck space infection, malignancy, and autoimmune processes. Given the exposure to the kitten and scratch history, CSD was suspected.

A fine-needle aspiration yielded purulent material, which was sent for Gram stain, bacterial culture, and acid-fast bacilli testing. The patient was started on intravenous clindamycin empirically pending culture results. After consultation with Infectious Diseases, oral azithromycin was initiated. Serology confirmed *B. henselae* infection (IgM = 1:80, IgG = 1:512). Cultures were negative. Within three weeks of azithromycin treatment, the patient had complete resolution of swelling and symptoms. No adverse effects occurred, and the patient returned to baseline health.

## Discussion

This case highlights the diagnostic complexity of persistent unilateral lymphadenopathy in pediatric patients, particularly when standard management for common causes such as Group A *Streptococcus* fails. While reactive lymphadenopathy is a frequent etiology, clinicians must broaden the differentials when symptoms persist or progress.

Differential diagnosis

Postauricular swelling can result from infectious, congenital, neoplastic, or autoimmune causes. Infectious agents include viruses (e.g., rhinovirus, adenovirus, influenza), typical bacteria (e.g., *Staphylococcus aureus*, *Streptococcus pyogenes*), atypical mycobacteria, and *B. henselae* [2]. Non-infectious causes include branchial cleft anomalies, lymphoma or other malignancies, and inflammatory conditions. In this case, failure to improve with a full antibiotic course and the evolution to an abscess necessitated expanded evaluation. The lack of cough, weight loss, other risk factors for tuberculosis, the negative QuantiFERON test, and the resolution of the swelling after commencing antibiotics ruled out tuberculosis.

Cat scratch disease: epidemiology and clinical features

CSD is a zoonosis usually caused by a cat or kitten scratch or bite [3]. Regional lymph node enlargement near the injection site occurs within 7 to 60 days [5]. Although the axilla is the most common site, lymphadenopathy in the head and neck is also frequent in children [5]. Suppurative lymphadenitis or abscess formation happens in a small percentage of cases [2,4]. While most infections resolve without intervention, atypical symptoms, such as deep tissue involvement or delayed treatment response, can make diagnosis more challenging [1]. Our case demonstrated that the abscess extended into the nearby muscles, as demonstrated on ultrasound and CT, which required antibiotic treatment.

Diagnostic approach

The diagnosis of CSD relies on clinical suspicion, exposure history, physical findings, and serologic confirmation [1]. The normal CRP blood levels in this case ruled out any systemic involvement. Imaging can identify abscess formation and exclude other pathologies. The normal mastoid bone and clear mastoid air cells on CT findings in our patient ruled out mastoiditis. Serologic testing remains the most accessible method for confirming *B. henselae* infection, though interpretation must consider potential cross-reactivity [2]. In our case, serology confirmed *B. henselae* infection with an IgM titer of 1:80 (reference range <1:20) and an elevated IgG antibody titer of 1:512 (reference range <1:64). IgM has a short response to *B. henselae* and may be negative at the time of testing. This can cause the infection to be missed. IgG >1:256 correlates with acute infection, and a low IgG antibody may suggest the onset or the end of the infection. When the IgG titers are between 1:64 and 1:256, a second serum sample is recommended to detect any titer increase that can confirm the diagnosis [2].

Management and outcomes

CSD has a good prognosis. Most uncomplicated CSD cases may resolve without antibiotics, but azithromycin is recommended in moderate-to-severe or suppurative cases and has been shown to shorten symptom duration [2]. For our patient, azithromycin was started after an Infectious Disease consultation and positive serology, leading to symptom resolution. Fine-needle aspiration was appropriate given the size and fluctuation of the lesion. Most immunocompetent children recover fully, though residual lymphadenopathy can persist for weeks.

## Conclusions

This case of persistent posterior auricular swelling caused by *B. henselae* reinforces the importance of considering CSD when evaluating prolonged unilateral lymphadenitis in children, especially with a lack of response to empiric antibiotics. History should include an inquiry about household pets, including contact with cats, especially kittens, whether in a neighbor’s yard, a friend’s house, or at the park. Careful physical examination of all areas drained by the affected lymph node is essential. *B. henselae* can present with a wide range of manifestations, with regional lymphadenopathy being the most common in immunocompetent children. Targeted treatment in cases that produce suppuration, such as this case, with the right antibiotics will lead to complete resolution of symptoms.
